# Effect of Vitamin D Supplementation on Oxidative Stress Biomarkers in Women Following Religious or Intermittent Fasting Patterns

**DOI:** 10.3390/nu17213389

**Published:** 2025-10-28

**Authors:** Spyridon N. Karras, Konstantinos Michalakis, Maria Kypraiou, Marios Anemoulis, Antonios Vlastos, Georgios Tzimagiorgis, Costas Haitoglou, Fotios Tekos, Zoi Skaperda, Periklis Vardakas, Neoklis Georgopoulos, Evangelos G. Papanikolaou, Demetrios Kouretas

**Affiliations:** 1 Laboratory of Biological Chemistry, Medical School, Aristotle University, 55535 Thessaloniki, Greece; tzimagio@auth.gr (G.T.); haitoglu@auth.gr (C.H.); 2 Endocrine Practice, Department of Obesity and Metabolism, 11521 Athens, Greece; kostismichalakis@hotmail.com; 3 Assisting Nature Centre of Reproduction and Genetics, 57001 Thessaloniki, Greece; mariabioanalysis@gmail.com (M.K.); papanikolaou@assistingnature.gr (E.G.P.); dkouret@gmail.com (D.K.); 4 Medical School, Aristotle University, 55535 Thessaloniki, Greece; mariosanemoulis@hotmail.com (M.A.); antonisvlastos1958@gmail.com (A.V.); 5 Department of Biochemistry and Biotechnology, School of Health Sciences, University of Thessaly, 41500 Larissa, Greece; fotis.tek@gmail.com (F.T.); zoiskap94@gmail.com (Z.S.); periklis_vardakas94@hotmail.com (P.V.); 6 Division of Endocrinology, Department of Internal Medicine, School of Health Sciences, University of Patras, 26504 Patras, Greece; neoklisgeorgo@gmail.com

**Keywords:** vitamin D, oxidative stress, TAC, GSH, TBARS, fasting, women, supplementation

## Abstract

Background: Vitamin D supplementation may influence oxidative stress, but evidence in populations following specific dietary patterns is limited. Methods: In this non-randomized, two-group exploratory study, 50 Orthodox nuns received vitamin D supplementation (2000 IU/day orally) for 16 weeks, whereas 50 age-matched women following time-restricted eating (TRE) served as controls receiving no supplementation. Anthropometric parameters, serum 25-hydroxyvitamin D [25(OH)D], and oxidative stress markers—total antioxidant capacity (TAC), glutathione (GSH), and thiobarbituric acid reactive substances—were measured at baseline and post-intervention. Results: At baseline, both groups were comparable in anthropometric and oxidative stress markers, except for serum 25-hydroxyvitamin D [25(OH)D], which was lower in the intervention group. Following supplementation, serum 25(OH)D increased from 15.77 ± 5.21 to 31.24 ± 7.87 ng/mL (*p* = 0.031) in Orthodox nuns. No significant changes were observed for TAC (0.93 ± 0.11 to 0.97 ± 0.09, *p* = 0.081) and GSH (6.01 ± 1.55 to 5.81 ± 1.41, *p* = 0.069), whereas TBARS decreased significantly (7.32 ± 1.31 to 6.94 ± 1.21, *p* = 0.041). No significant changes were observed in controls under TRE. Changes (Δ) in all variables represented the post–pre difference over the 16-week period. Pearson correlations showed no significant associations between Δ25(OH)D and ΔTAC (r = −0.244, *p* = 0.346), ΔGSH (r = 0.110, *p* = 0.675), or ΔTBARS (r = −0.116, *p* = 0.657). In multivariable regression adjusted for age, weight, body fat percentage, and baseline 25(OH)D, Δ25(OH)D was not an independent predictor of oxidative stress marker changes; however, weight (β = 0.08, *p* = 0.011) and body fat percentage (β = −0.13, *p* = 0.014) were associated with reductions in TBARS. Conclusions: In conclusion, sixteen weeks of vitamin D supplementation in women adhering to Orthodox fasting produced no consistent improvements in oxidative stress markers. While a small reduction in TBARS was observed, this effect was modest and appeared indirect, being more closely associated with decreases in body weight and fat mass than with vitamin D status itself. Taken together, our findings indicate an overall neutral impact of vitamin D on redox balance, suggesting that any antioxidant benefit is likely secondary to metabolic or adiposity-related changes.

## 1. Introduction

Vitamin D is a lipophilic secosteroid classically linked to calcium and skeletal balance, yet accumulating evidence highlights wider actions, ranging from immune modulation to the regulation of redox pathways [1]. Oxidative stress occurs when the generation of reactive oxygen species (ROS) surpasses the body’s antioxidant defenses, a state implicated in the development of cardiovascular, metabolic, and neoplastic disorders [2]. There is growing interest in understanding how nutritional interventions may influence oxidative stress, particularly in populations adhering to alternative dietary patterns such as religious or intermittent fasting [3].

Both Christian Orthodox fasting (COF) and time-restricted eating (TRE) constitute highly structured nutritional patterns that may influence oxidative balance and metabolic regulation. The COF regimen involves abstinence from animal products and consumption of plant-based foods within a defined time window [4,5], while TRE often imposes a daily feeding schedule within 8 h [6]. These dietary practices may lead to alterations in energy intake, macronutrient composition, and antioxidant consumption, ultimately affecting redox status. Furthermore, both regimens may reduce inflammation and improve metabolic markers [7,8]. While several studies have examined the impact of fasting on glucose and lipid metabolism [9], less is known about its interaction with vitamin D metabolism and oxidative stress [10].

Emerging evidence suggests that vitamin D may act as a modulator of oxidative stress through several biological pathways. Experimental studies indicate that vitamin D can reduce ROS generation by inhibiting NADPH oxidase activity, enhance mitochondrial function, and upregulate antioxidant defense systems such as glutathione synthesis [11,12]. Observational data also support an association between low vitamin D levels and increased oxidative damage, although results are not always consistent, highlighting the need for controlled intervention studies [13,14].

In parallel, fasting and plant-based dietary patterns may influence both vitamin D status and oxidative balance. Reduced intake of animal products, seasonal variations in sun exposure, and differences in nutrient composition can predispose individuals adhering to fasting regimens to vitamin D deficiency. At the same time, higher consumption of antioxidant-rich foods such as legumes, fruits, and vegetables could independently modulate redox status. These complex and potentially opposing effects make it particularly relevant to explore whether vitamin D supplementation during fasting can provide measurable benefits in oxidative stress regulation.

Vitamin D deficiency has been linked to increased oxidative damage through mechanisms involving NADPH oxidase inhibition and glutathione regulation [11,12,13,14]. Thus, vitamin D supplementation during fasting could theoretically attenuate oxidative damage and enhance antioxidant defense.

The present study investigates the effect of 16-week vitamin D supplementation on key oxidative stress markers—TAC, GSH, and TBARS (oxidative stress markers)—in Orthodox nuns compared to a control non-supplemented group of women following TRE dietary patterns for promoting health. We aimed to determine whether vitamin D affects redox homeostasis and to assess whether baseline vitamin D status influences this response.

## 2. Methods

### 2.1. Study Design

This was a prospective non-randomized 16-week intervention trial of COF and TRE in two groups of adult female nuns and lay women. We included a group of Orthodox nuns from monasterial communities of Northern Greece, which received a 2000 IU daily dose of vitamin D_3_ in the form of oral soft gel capsules for 16 weeks, and a control group of lay women did not receive any form of vitamin D supplement, during the study period, following only a TRE dietary regimen. Lay women following time-restricted eating (TRE) were chosen as controls because both regimens share structured eating windows and potential caloric restriction, allowing comparison between two fasting patterns differing mainly by vitamin D supplementation.

### 2.2. Participants

We included 50 Christian Orthodox female adult nuns, from two different monasteries, 30–50 years of age, residing in Central and Northern Greece and an age-matched cohort of 50 adult lay women from the same region. Orthodox nuns (but not lay women), with a baseline 25-hydroxyvitamin D concentrations ≥ 20 ng/mL (as initially evaluated from the same initial cohort—results published previously [15,16,17,18] were excluded. Women from the control group followed TRE dietary patterns at least for the last year for health promoting reasons. Additional exclusion criteria for both groups were the following: Body mass index (BMI) ≤ 25, amenorrhea ≥ 3 months, pregnancy, administration of medications that can alter weight, glucose and lipid metabolism.

### 2.3. Intervention

Women living in Orthodox monasteries who had consistently practiced Christian Orthodox fasting (COF) for at least 16 weeks were recruited, while women from the general population participated in a 16-week program of time-restricted eating (TRE) following a 3-week wash-out phase. The nuns adhered to the Athonian fasting model that has been previously documented [19,20,21,22], whereas the control group from the general population consumed a diet that permitted low-fat meat products, without strict regulation of macronutrient composition or total caloric intake. In addition, the fasting group of nuns followed a fixed eating window of eight hours (08:00–16:00), as dictated by monastery dietary rules, while the control group followed a TRE regime and consumed food from 09:00 to 17:00. Adherence to dietary plans was evaluated with a 3-day food record (two weekdays and one weekend day) at the end of the study period. Dietary records were evaluated using the Food Processor Nutrition Analysis Software 2023 (ESHA Research, https://esha.com/products/food-processor/, accessed on 2 August 2024) [23]. In parallel, data on physical activity were collected for all participants, including frequency, duration, and intensity, and categorized into light, moderate, and vigorous exercise according to the American Heart Association guidelines [24]. Dietary intake, data on physical activity were reported previously [3]. In our previous publication [3] we focused on baseline data describing vitamin D status, fasting patterns, and general lifestyle parameters. The current article presents new analyses based on the intervention phase of that cohort, including oxidative stress biomarkers.

### 2.4. Anthropometric Measurements and Biochemical Analysis

Anthropometric and biochemical assessments were carried out in both study groups using standardized protocols. Detailed descriptions of the methods, reference ranges, and equipment have been published previously [25,26,27,28]. Serum calcium (Ca) was quantified with the COBAS 8000 automated analyzer system (Roche Diagnostics GmbH, Mannheim, Germany). Parathyroid hormone (PTH) and 25-hydroxyvitamin D [25(OH)D] were measured with the COBAS e 602 immunochemistry module, employing electro-chemiluminescence (ECL) technology (Roche Diagnostics GmbH, Mannheim, Germany). Biomarkers of oxidative stress were determined with validated assays that have been previously reported in detail [3,29,30,31,32].

### 2.5. Ethical Considerations

All participants provided written informed consent prior to enrollment in the study.

### 2.6. Statistical Analysis

Continuous variables are presented as mean ± SD. Paired-samples *t*-tests were used for comparisons of dietary and nutrient intake within groups. Differences in age across physical activity categories (light, moderate, intense) were assessed with one-way ANOVA followed by Tukey’s post hoc test. The association between physical activity levels and health markers was evaluated using ANCOVA with age as a covariate. Normality was examined with the Shapiro–Wilk test. Parametric tests were applied when assumptions were met; otherwise, non-parametric alternatives or variable transformations were used. Regression model residuals were checked to confirm model validity. Between-group differences were evaluated with the Mann–Whitney U test. Multivariable associations were examined with linear regression models. For all analyses, statistical significance was set at *p* < 0.05 (two-sided). Analyses were performed with SPSS, version 22 (IBM Corp., Armonk, NY, USA).

## 3. Results

Orthodox nuns were older than lay women (median age 42 ± 5.8 vs. 38 ± 2.9, *p* < 0.015) but did not differ in median weight and BMI. At baseline, both groups were comparable in terms of anthropometric and oxidative stress markers, except for vitamin D concentration, which were lower in the intervention group. Following the 16-week vitamin D supplementation, the Orthodox nuns exhibited a significant increase in serum 25(OH)D levels (from 15.77 ± 5.21 to 31.2 ± 7.87 ng/mL, *p* < 0.001). Body weight, BMI, and body fat percentage showed modest but statistically significant reductions in the intervention group (all *p* < 0.05), while no changes were observed in the control group. As for oxidative stress markers, non-significant changes were observed, with the exception of a slight TBARS reduction. Specifically, TAC increased (from 0.93 ± 0.11 to 0.97 ± 0.09, *p* = 0.081), whereas concentrations of GSH and TBARS declined (6.01 ± 1.55 to 5.81 ± 1.41, *p* = 0.069) and (7.32 ± 1.31 to 6.94 ± 1.21, *p* = 0.041) accordingly, following supplementation in the intervention group, without significant changes (Table 1). No significant differences were also observed in the control group across any of these markers.

**Figure 1:**
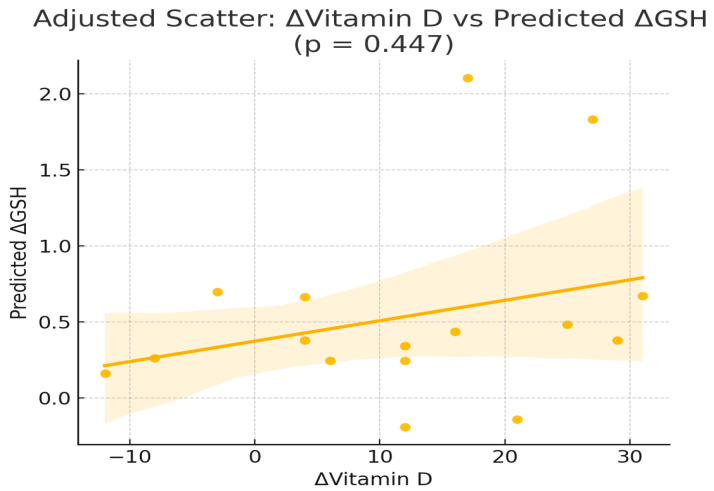
Scatterplot of the variation in serum 25-hydroxyvitamin D [Δ25(OH)D] compared with the variation in glutathione (ΔGSH) in the intervention group (Orthodox nuns). (r = 0.110, *p* = 0.675).

To explore whether changes in serum vitamin D concentrations were independently correlated or predicted changes in oxidative stress markers, we performed correlation and multivariable linear regression models in the intervention group (Orthodox nuns), adjusting for age, body weight, body fat percentage, and baseline 25(OH)D concentrations (Table 2 and Table 3). Pearson correlation analysis in the intervention group (Orthodox nuns) showed no significant linear associations between the change in serum 25-hydroxyvitamin D [Δ25(OH)D] and changes in TAC (r = −0.244, *p* = 0.346), GSH (r = 0.110, *p* = 0.675), or TBARS (r = −0.116, *p* = 0.657) (Table 2, Figure 1).

**Figure 2:**
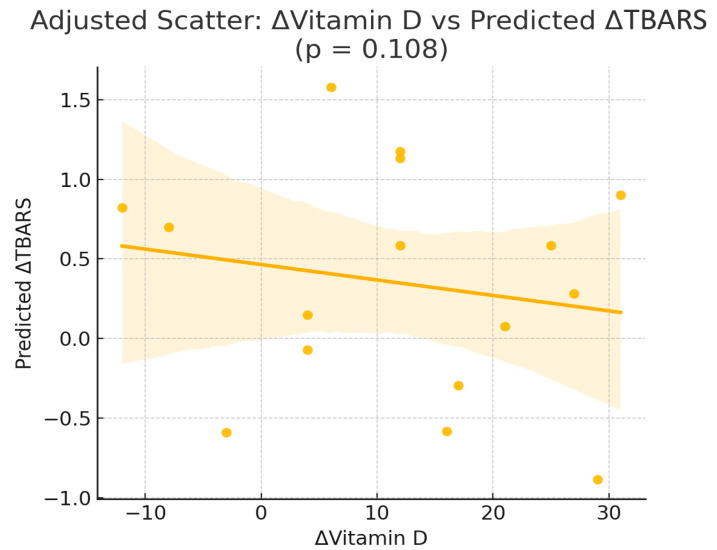
Scatterplot of the Δ25(OH)D levels compared with the shift in thiobarbituric acid reactive substances (ΔTBARS) in the intervention group (Orthodox nuns). (r = −0.116, *p* = 0.657).

In multivariable linear regression models adjusted for age, weight, body fat percentage, and baseline 25(OH)D (Table 3), Δ25(OH)D did not emerge as an independent predictor of ΔTAC, ΔGSH, or ΔTBARS (Figure 2). For ΔGSH, none of the included predictors reached statistical significance. For ΔTAC, no variables were significantly associated with changes in antioxidant capacity. In contrast, for ΔTBARS, both weight (β = 0.08, *p* = 0.011) and body fat percentage (β = −0.13, *p* = 0.014) were independently associated with reductions in lipid peroxidation.

Subgroup analysis by baseline 25(OH)D levels (<20 ng/mL vs. ≥20 ng/mL) showed no significant differences in the post-supplementation changes in TAC, GSH, or TBARS.

## 4. Discussion

Vitamin D is a multi-potent factor that was initially considered as a vitamin but is gradually being recognized as a hormone affecting multiple states of the body, apart from skeletal effects. Vitamin D exerts various roles on metabolic parameters, as well as oxidative stress, whose reduction is beneficial on endothelial and cardiac function [33,34,35].

When the body’s oxidative processes outweigh its antioxidant defenses, which should normally be in balance, oxidative stress causes cellular damage through the activity of reactive oxygen species (ROS). Glutathione peroxidase, superoxide dismutase, and other vitamins, including vitamin D, are all part of the antioxidant defense system. In particular, vitamin D has anti-oxidant properties by positively regulating superoxide dismutase and glutathione in cells [2].

Recent research has also focused on TRE and its possible positive impacts on cardiometabolic health. A calorie-restricting diet and an intermittent-fasting diet, which limits feeding times during specific hours, have different levels of metabolic and stress hormones [10]. Measurements of inflammation, oxidative stress, and cardiometabolic health hormones and cytokines (insulin, ghrelin, leptin, glucagon, adiponectin, resistin, advanced glycated-end products (AGE), advanced oxidation protein products, total ni-trite-nitrate levels, tumor necrosis factor-α, interleukin (IL)-6, IL-8, and IL-10) were made in order to examine the effects of intermittent fasting and demonstrated that feeding under time restriction led to notable decreases in AGEs (approximately 25%) and advanced oxidation protein products (approximately 31%) [25]. The findings of this study demonstrate that short-term vitamin D supplementation in Orthodox nuns adhering to religious fasting patterns had no effects in improving oxidative status under COF compared to TRE, with the exception of a potential subtle effect on TBARS concentrations. These results suggest a neutral effect of vitamin D on oxidative stress markers in a unique population adhering to restrictive dietary and lifestyle regimens.

Markers of lipid peroxidation, expressed as TBARS concentrations, showed a significant decline after vitamin D supplementation [36,37], decreased significantly following vitamin D supplementation. This finding aligns with previous studies showing antioxidant effects of vitamin D through mechanisms that suppress reactive oxygen species [38,39] and downregulate pro-oxidant enzymes such as NADPH oxidase [40,41]. TBARS levels, which reflect lipid peroxidation and serve as a general index of oxidative stress [36,37], decreased significantly following vitamin D supplementation. This finding aligns with previous studies showing antioxidant effects of vitamin D through mechanisms that suppress reactive oxygen species [38,39] and downregulate pro-oxidant enzymes such as NADPH oxidase [40,41]. Mechanistically, vitamin D may exert antioxidant effects through modulation of intracellular redox pathways. Evidence suggests that vitamin D receptor activation can inhibit NADPH oxidase activity, thereby reducing ROS generation and lipid peroxidation [40,41]. In addition, vitamin D influences glutathione homeostasis by promoting the synthesis and recycling of reduced glutathione (GSH), a key cellular antioxidant [11,14,42,43,44]. Such mechanisms could theoretically protect against oxidative damage in fasting individuals, although in our study the magnitude of change in GSH was limited, highlighting that these pathways may only partly explain the observed trends.

Another pathway linking vitamin D to oxidative stress relates to adiposity and metabolic regulation. Adipose tissue is a significant source of ROS, and weight loss is consistently associated with improvements in oxidative balance [45,46]. Vitamin D has been proposed to attenuate adipose tissue-driven oxidative stress through effects on inflammation and adipogenesis [2,38,39]. In our cohort, reductions in TBARS were observed in parallel with decreases in body weight and fat mass, suggesting that any antioxidant benefits of vitamin D may have been mediated indirectly via changes in adiposity, rather than reflecting a direct effect on redox enzymes alone.

Interestingly, our subgroup analysis found no differential response in oxidative stress markers based on baseline vitamin D status (<20 ng/mL vs. ≥20 ng/mL). This suggests that the antioxidative effects of vitamin D may occur regardless of deficiency thresholds and supports its pleiotropic action across a range of vitamin D levels. Furthermore, no significant changes were observed in the control group, supporting the conclusion that observed shifts in oxidative stress biomarkers are attributable to the supplementation rather than time of feeding or fasting alone. In addition, this pilot study provides evidence for the effects of TRE for 16 weeks, demonstrating no effects on oxidative equilibrium. It is important to note that participants in the intervention group also experienced reductions in body weight and fat mass, which are known as crucial modulators of oxidative stress [45,46].

The observed small reduction in TBARS was not directly associated with serum vitamin D changes. Instead, it appeared to be more strongly related to alterations in body weight and fat mass, in line with previous studies reporting that improvements in TBARS are often linked to changes in body composition rather than micronutrient supplementation per se. The small decline in TBARS likely reflects concurrent improvements in body composition rather than a direct antioxidative action of vitamin D itself. Thus, our findings suggest that vitamin D might have only an indirect role, possibly mediated through effects on adiposity.

While these findings are promising, limitations should be acknowledged. A key limitation of our study is the absence of randomization and the use of a non-equivalent control group (nuns vs. lay women). This design may have introduced confounding, particularly given the baseline differences in vitamin D status between the two groups. While this reduces the strength of causal inference, it should be noted that the monastic population offers a rare opportunity to study women with long-term adherence to Orthodox fasting, which provides valuable though preliminary insights.

The relatively small sample size may limit statistical power for subgroup or interaction effects. Moreover, the study was not randomized, and although both groups followed structured fasting protocols, residual confounding may persist. Another limitation of our study is that dietary intake of antioxidants was not assessed. This represents a potential confounder, as antioxidant-rich foods may influence oxidative stress markers independently of vitamin D supplementation. Future studies should incorporate validated dietary assessment tools on this setting.

Oxidative stress biomarkers used provide only a partial picture of the redox system, and further studies should explore additional markers such as catalase, superoxide dismutase, or 8-isoprostane [47]. An additional limitation of the present study is the relatively short intervention period of 16 weeks, which may not have been sufficient to capture the full extent of potential changes in oxidative stress biomarkers, particularly for parameters that respond more slowly to nutritional interventions. Furthermore, the study population consisted exclusively of middle-aged women adhering to specific religious dietary practices, which may limit the generalizability of the findings to other populations with different lifestyles, age ranges, or health statuses. Lastly, the lack of dietary intake assessment for antioxidant-rich foods or other supplements prevents us from fully accounting for potential dietary confounders that could have influenced oxidative stress outcomes. Although our findings suggested a modest reduction in TBARS levels following vitamin D supplementation, these results should be interpreted with caution. The effect size was small and appeared more strongly associated with changes in body weight and fat mass rather than with vitamin D status itself. Taken together, these limitations indicate that the observed effects may reflect indirect or confounded associations rather than a direct causal link between vitamin D and oxidative stress.

Finally, given that all participants were residents of Greece, a southern-European population with typically lower baseline 25(OH)D concentrations, the present findings may not be generalizable to populations with different genetic backgrounds or vitamin D metabolism profiles [48,49,50,51,52,53,54,55,56].

## 5. Conclusions

In conclusion, the findings of the present study indicate that 16 weeks of vitamin D supplementation in women following an Orthodox religious fasting regimen had an overall neutral effect on oxidative stress markers. Although a small but statistically significant reduction in TBARS levels was observed, the other markers (TAC, GSH) showed no significant changes. Multivariable regression analysis did not identify the change in vitamin D status as an independent predictor of oxidative stress marker variation, while reductions in TBARS were mainly associated with changes in body weight and body fat percentage. Taken together, the present findings suggest that vitamin D supplementation exerts at most a marginal and possibly indirect effect on oxidative stress markers in women adhering to Orthodox fasting, with changes more likely linked to alterations in body composition. These results should therefore be interpreted with caution, and no causal inference can be drawn from this study design. Τhe impact of vitamin D supplementation on the oxidative profile of this specific population is limited and potentially secondary to body composition changes. Because this was a non-randomized, non-equivalent group design, causal inference is limited; therefore, the present work should be considered a pilot exploratory study. Future randomized trials should include quantitative assessment of dietary antioxidant intake (e.g., vitamins C, E, and polyphenols) to better isolate the specific contribution of vitamin D supplementation to oxidative stress modulation.

Our findings indicate only a limited and potentially neutral role of vitamin D in redox regulation within this population. Future studies using randomized controlled designs, with standardized dietary assessment and improved methodological rigor, are warranted to confirm or refute these preliminary observations.

## Figures and Tables

**Table 1:** Anthropometric and oxidative stress parameters before and after vitamin D supplementation in Orthodox nuns (intervention group) and in controls. Values are presented as mean ± SD. *p*-values correspond to within-group comparisons (baseline vs. post-intervention) using paired-samples *t* tests. Between-group differences were assessed separately with Mann–Whitney U tests and are described in Section 3.

Variable	Supplementation-Baseline	Supplementation-Post	*p*	Controls-Baseline	Controls-Post	*p*
Weight (kg)	71.52 ± 9.85	70.16 ± 9.51	0.021	68.75 ± 8.62	69.6 ± 8.29	0.287
BMI (kg/m^2^)	27.02 ± 3.96	26.47 ± 3.75	0.194	26.53 ± 3.54	26.8 ± 3.54	0.476
Body fat (%)	34.71 ± 8.42	33.96 ± 8.23	0.163	33.12 ± 7.97	32.6 ± 7.93	0.312
25(OH)D (ng/mL)	15.77 ± 5.21	31.24 ± 7.87	0.031	26.41 ± 7.56	28.9 ± 7.58	0.534
TAC	0.93 ± 0.11	0.97 ± 0.09	0.081	0.79 ± 0.08	0.79 ± 0.08	0.267
GSH	6.01 ± 1.55	5.81 ± 1.41	0.069	7.11 ± 1.74	6.74 ± 1.74	0.453
TBARS	7.32 ± 1.31	6.94 ± 1.21	0.041	7.4 3 ± 1.11	7.26 ± 1.12	0.634

**Table 2:** Pearson correlation coefficients between changes in serum 25-hydroxy-vitamin D (ΔVitamin D) and changes in oxidative stress markers (ΔTAC, ΔGSH, ΔTBARS) following vitamin D supplementation in Orthodox nuns.

Outcome	Pearson r	95% CI	*p*-Value
TAC	−0.244	−0.58 to 0.237	0.3463
GSH	0.11	−0.435 to 0.64	0.6748
TBARS	−0.116	−0.535 to 0.318	0.6572

**Table 3:** Multivariable linear regression models predicting changes in oxidative stress markers (Δ_TAC, Δ_GSH, Δ_TBARS) based on ΔVitamin D, adjusted for age, weight, body fat percentage, and baseline 25(OH)D. Each row displays the coefficient (β), standard error, and *p*-value for each predictor. * For all analyses, statistical significance was set at *p* < 0.05 (two-sided).

Outcome	Variable	Coef.	Std. Err.	*p*-Value
ΔGSH	const	−1.11	4.58	0.814
ΔGSH	ΔVitD	0.05	0.06	0.447
ΔGSH	AGE (y)	0.01	0.05	0.866
ΔGSH	WEIGHT (kg)	−0.0	0.06	0.948
ΔGSH	BODY FAT %	−0.02	0.11	0.856
ΔGSH	25(OH)-D3 (ng/mL)	0.06	0.07	0.375
ΔTBARS	const	−1.18	1.89	0.548
ΔTBARS	ΔVitD	−0.05	0.03	0.108
ΔTBARS	AGE (y)	0.03	0.02	0.128
ΔTBARS	WEIGHT (kg)	0.08	0.02	0.011 *
ΔTBARS	BODY FAT %	−0.13	0.05	0.014 *
ΔTBARS	25(OH)-D3 (ng/mL)	−0.03	0.03	0.246
ΔTAC	const	−0.21	0.11	0.087
ΔTAC	ΔVitD	0.0	0.0	0.991
ΔTAC	AGE (y)	0.0	0.0	0.107
ΔTAC	WEIGHT (kg)	0.0	0.0	0.244
ΔTAC	BODY FAT %	−0.0	0.0	0.999
ΔTAC	25(OH)-D3 (ng/mL)	0.0	0.0	0.966

## Data Availability

The original contributions presented in this study are included in the article. Further inquiries can be directed to the corresponding author.
